# New Melissiodontinae (Mammalia, Rodentia) from the Paleogene of south-east Serbia

**DOI:** 10.1007/s12549-017-0311-2

**Published:** 2018-01-27

**Authors:** Wilma Wessels, Andrew A. van de Weerd, Hans de Bruijn, Zoran Marković

**Affiliations:** 1 Department of Earth Sciences, Utrecht University, Princetonlaan 8A, 3584 CB Utrecht, The Netherlands; 2 Natural History Museum in Belgrade, Njegoševa 51, Belgrade, 1100 Serbia

**Keywords:** Paleogene, Rodentia, Melissiodontinae, New genus and species, South-east Serbia

## Abstract

Isolated teeth of Melissiodontinae from two Eocene and four Oligocene localities in southeastern Serbia are described. One new genus and two new species are named. The study of the derived morphology of the cheek teeth and of the contrastingly primordial microstructure of the tooth enamel of this diverse material provides a glimpse into the early history of the subfamily. The supposedly Asian murid ancestor of the Melissiodontinae seems to have reached the Serbian-Macedonian land area during the early or middle Eocene, which is shortly after the split up of the Muridae and Dipodidae and before the ‘Grande Coupure’ of central and Western Europe. We interpret the rapid consequent specialisation of the morphology of the chewing apparatus of the Melissiodontinae as an adaptation to feeding on small invertebrates on the floor of the Eocene forest.

## Introduction

The subfamily Melissiodontinae is in Europe represented by only one genus*: Melissiodon* Schaub, 1925*,* known from fossil assemblages dated between the late early Oligocene (MP23) to early Miocene (MN4) and includes nine species (Hrubesch 1957). It is the only murid that is present during the ‘Cricetid Vacuum’ (MN3 in the early Miocene) in south western Europe. Outside Europe, *Melissiodon* sp. is known from the Anatolian assemblage of Kargı-1 dated latest Oligocene-earliest Miocene (de Bruijn et al. 2013). The other genus belonging in this subfamily, *Edirnella* Ünay-Bayraktar, 1989, is known from the late Eocene of Süngülü (Lesser Caucasus; de Bruijn et al. 2003) and from two localities in the Thrace basin (MP25, Ünay-Bayraktar 1989).

The common presence of the Melissiodontinae in almost all of our Serbian localities was unsuspected. Moreover, the morphological diversity observed indicate that the Melissiodontinae were already diverse and highly specialised in the late Eocene and early Oligocene in this area (Table 1).

**Table 1:** Distribution of the rodent species in the localities of S.E. Serbia based on the total number of first and second molars

			Eocene		Early Oligocene					
Family	Subfamily	Genus and species	Zvonce	Buštranje	Strelac-1	Strelac-2	Strelac-3	Valniš	Raljin	Total M1–M2
Diatomyidae	Diatomyinae	*Inopinatia balkanica*			7	4	3	49	2	65
Dipodidae	primordial Zapodidae	*Heosminthus borrae*				X	22	20	1	43
Muridae	Pseudocricetodontinae	*Heterocricetodon* nov. sp. A			14	5	6	49	4	78
		*Pseudocricetodon* nov. sp. (small)		29			14			43
		*Pseudocricetodon montalbanensis*			4		23	28	8	63
	Paracricetodontinae	*Paracricetodon dehmi*			3		X	10		13
		*Paracricetodon* nov. sp. B					2	11	?	13
		*Paracricetodon* nov. sp. A		75	45	26	30	127	5	308
	Pappocricetodontinae	*Witenia* sp.			5		X	2		7
		nov. gen. 3 nov. sp. A		601						601
		*Witenia* n. sp. A		21						21
	Melissiodontinae	cf. *Edirnella* nov. sp. 2			6	1				7
		*Mogilia lautus* n.gen. n.sp.			X			34	1	35
		*Mogilia miloshi* gen. n.sp.	28	30						58
		cf. *Edirnella* nov. sp. 1		4						4
		cf. *Edirnella* sp. indet.	X							1
	?Spalacinae	nov. gen. 1 sp. A	3							3
Total number of upper and lower M1 and M2 in each locality			31	760	84	36	100	330	21	1363

The samples of isolated teeth of melissiodontines were collected in the Babušnica-Koritnica and Pčinja basins from two Eocene localities, Zvonce and Buštranje, and from five early Oligocene sites of Strelac-1, -2 and -3 Valniš and Raljin. The geological settings of these sites, their age assignment, overall fossil content and methods of sample treatment is described in de Bruijn et al. (in press).

## Material and methods

**Fig. 1:**
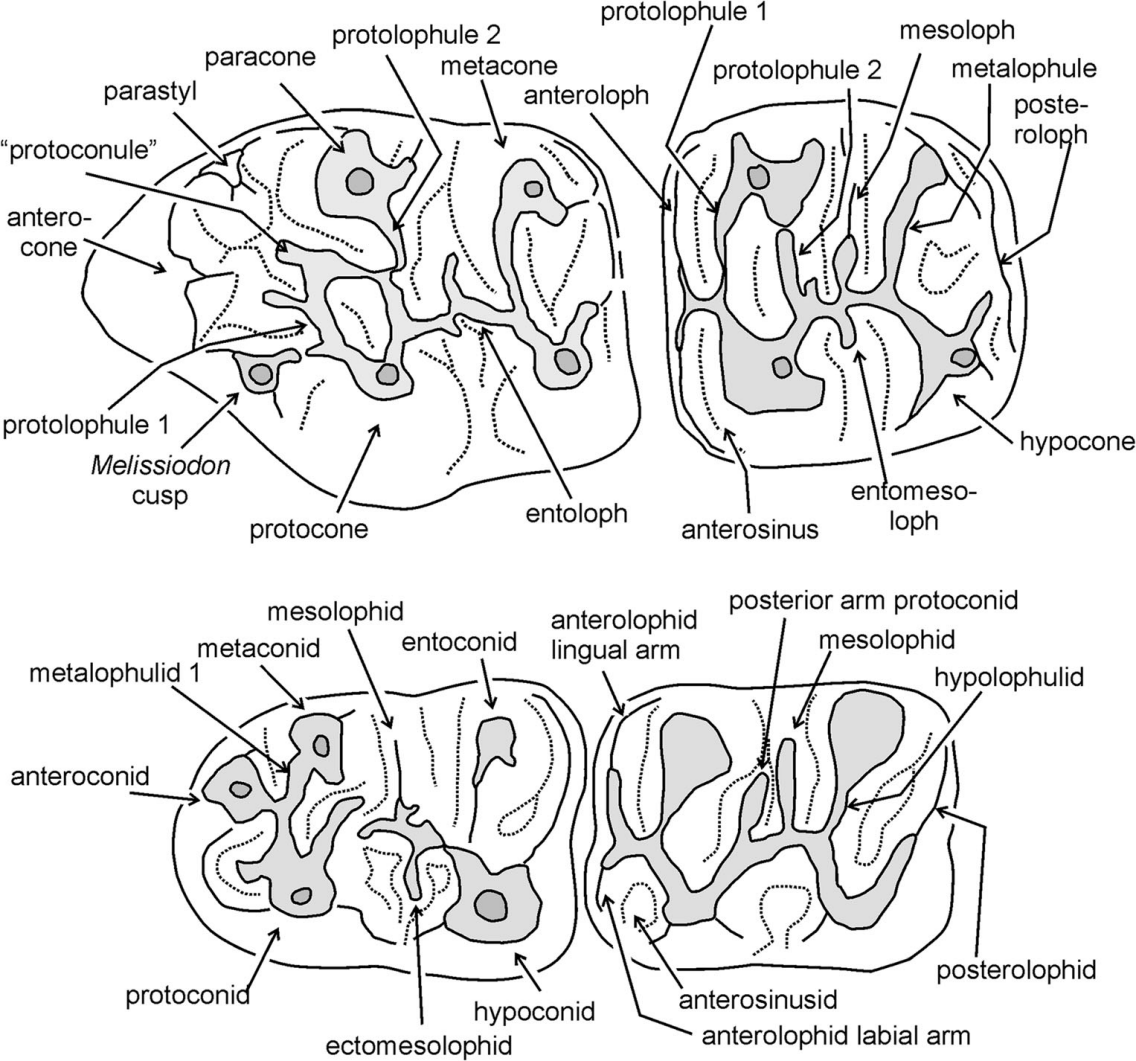
Terminology of elements of the molars of melissiodonts used in the descriptions

The terminology of parts of the cheek teeth basically follows Freudenthal et al. (1994) and is illustrated in Fig. 1. The melissiodontine material used for comparison consists of casts of *Edirnella sinani* Ünay-Bayraktar, 1989 cheek teeth and a fragment of a lower incisor from Kocayarma (Thrace basin, Turkey). In addition, casts of the cheek teeth of *Edirnella kempeni* de Bruijn et al. 2003 from Süngülü (Lesser Caucasus, Turkey) and original specimens of *Melissiodon bernlochensis* Hrubesch, 1957 from Bernloch (Germany) are available.

Abbreviations and terminology used in the description of the microstructure of enamel are enamel dentine junction (EDJ), Hunter-Schreger band (HSB), angle between the HSB and the normal to the EDJ (inclination), portio interna (PI), portio externa (PE), outer enamel surface (OES), external enamel layer without prims (PLEX), inter prismatic matrix (IPM), enamel with parallel prisms that are at right angles to the EDJ (radial enamel) and basal ring of lamellar enamel in the molars (BRLE). The measurements of the teeth have been taken with a Leitz Ortholux measuring microscope with mechanical stage and measuring clocks. The pictures were made using a table-top and a high-resolution SEM. All specimens are figured as left ones. If the original is from the right side, this is indicated by underlining its number on the figure. Lower case letters refer to the lower dentition, upper case letters refer to the upper dentition. Abbreviations for measurements and descriptions are number of specimens (*N*), range of measurements (*R*), length (*L*), width (*W*), sinistral (sin) and dextral (dex).

The abbreviations used for of the localities are Zvonce (ZV), Buštranje (BUS), Strelac-1 (STR-1), Strelac-2 (STR-2), Strelac-3 (STR-3), Valniš (VA) and Raljin (RA). The fossil assemblages from southeastern Serbia are housed in the Natural History Museum in Belgrade (Serbia). Belgrade Museum locality codes are ZV = 037, BUS = 031, STR-1 = 024, STR2 = 015, STR3 = 026, VA = 027, RA = 028. A representative set of casts of rodents is kept in the collection of the department of Earth Sciences of Utrecht University, the Netherlands.

## Taxonomy

Muridae Illiger, 1811

Melissiodontinae Schaub, 1925

**Genera included:** *Melissiodon* Schaub, 1920; *Edirnella* Ünay-Bayraktar, 1989 and *Mogilia* nov. gen.

### Introduction

Schaub (1920) defined the genus *Melissiodon* on the combination of characteristics of the mandible and teeth that differentiate this genus from all other murids. In the same publication, he remarked that the group merits family rank, but the formal definition of the Melissiodontidae was published 5 years later (Schaub 1925). It is clear from these publications, as well as from a number of later studies (i.e. Freudenthal et al. 1992), that specialists hesitated to include *Melissiodon* into the Muridae (the family name Muridae is used here as the equivalent of Muroidea). This is understandable because it shows a number of aberrant characteristics in the skull: the lower incisor ends below the m2, the diastema is long and tubular, the scar of the masseter on the mandible is weak, the infraorbital foramen is large and the shape of the jugal is not like in other murids (Kristkoiz 1992). At the same time, the morphology of the cheek teeth is very derived, while the microstructure of the lower incisors remained primitive (Kalthoff 2000). Allocation to the Muridae was therefore rather induced by the lack of a suitable alternative than on similarity to members of that family. All species of *Melissiodon* of which the characteristics of skull and mandible are known share the characteristics listed above as well as characteristics of the cheek teeth such as the high ridges with steep walls (Hrubesch 1957), laterally compressed cusps and a square occlusal surface of the M2. Consequently the contents of the (sub)family remained clear cut and restricted to *Melissiodon* until Ünay-Bayraktar (1989) allocated her genus *Edirnella*, which is based on a few isolated upper cheek teeth of *E. sinani* from Kocayarma (Thrace basin, Turkey; ~MP 25), to the Melissiodontidae.

The classification of the Oligocene Muridae as suggested by Ünay-Bayraktar (1989), in which the subfamilies Paracricetodontinae (with *Paracricetodon* and *Trakymys*) and Melissiodontinae (with *Melissiodon* and *Edirnella)* are united in the family Melissiodontidae, has been criticised by Freudenthal et al. (1992), Kristkoiz (1992) and Kalthoff (2006) on two issues: (1) is *Edirnella* a member of the Melissiodontinae? (2) Are the Paracricetodontinae and Melissiodontinae as closely related as suggested by Ünay-Bayraktar (1989)?

**Fig. 2:**
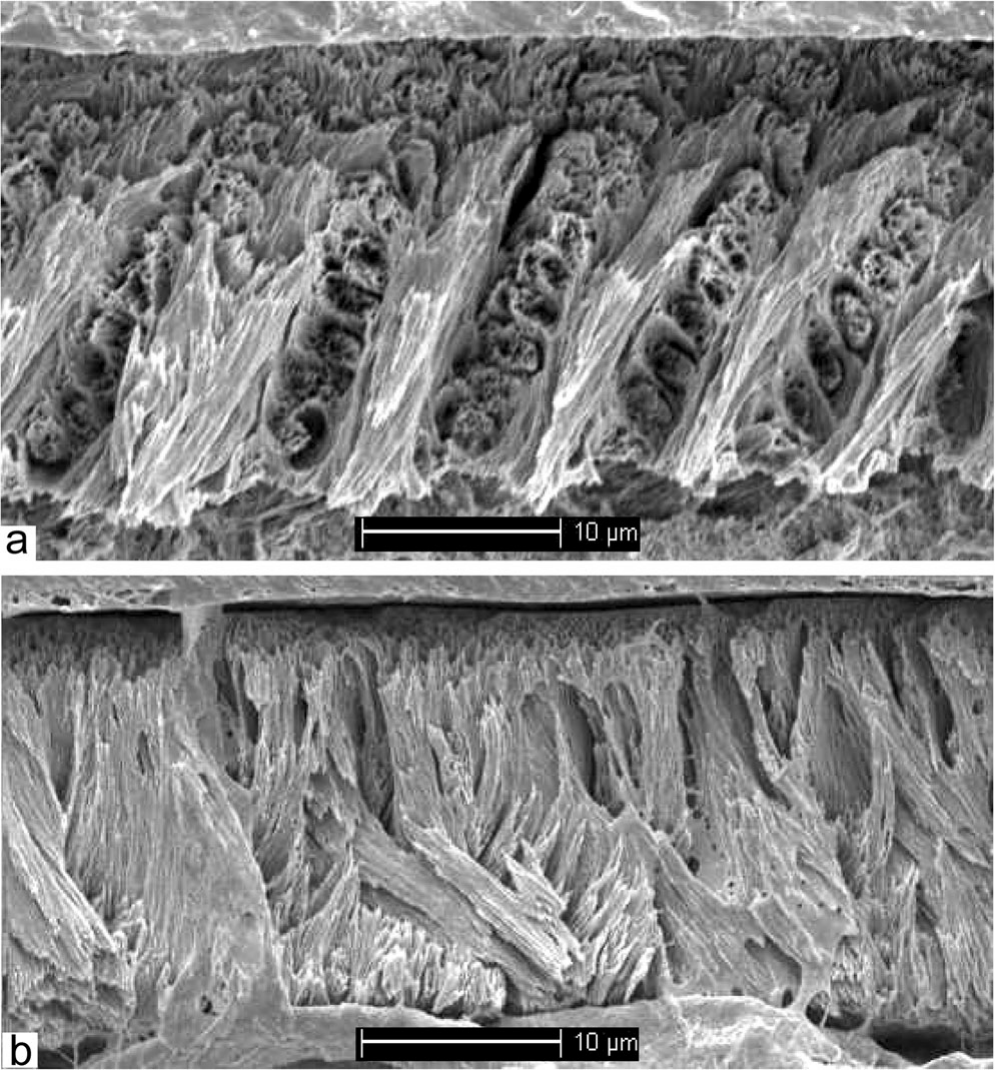
Longitudinal (a) and transverse (b) section of the lower incisor enamel of *Edirnella sinani* from its type locality Kocayarma (Turkish Thrace basin). The outside of the thin enamel (~ 10 mu) shows a set of 5–6 very fine parallel ridges. The relative to the portio externa (PE) very thick portio interna consists of transverse Hunter-Schreger (HSB) bands with prism parallel inter prismatic matrix (IPM). The angle between the HSB and the normal on the enamel dentine junction (EDJ) is ~ 15°. The thin portio externa consists of radial enamel. A plex is absent

Freudenthal et al. (1992) transferred *Edirnella* to the Paracricetodontinae on the basis of the morphology of its cheek teeth, while Kalthoff (2006) did so on the basis of the schmelzmuster of the lower incisor. However, the incisor studied by Kalthoff (2006) was erroneously identified as *Edirnella* from Kavakdere (Thrace basin, Turkey), a locality that did not yield this species. Since this mistake was made, as well as discovered, by one of us (HdB) we apologise for causing confusion. Our recent analyses of a lower incisor of *E. sinani* from its type locality Kocayarma showed that it has the primitive type 1 schmelzmuster (Fig. 2) characteristic for melissiodontines (Kalthoff 2000).

The recognition that the enamel of the lower incisor of *E. sinani* shows the type 1 schmelzmuster in combination with the morphology of the upper cheek teeth, justifies the classification of *Edirnella* and *Melissiodon* in the same subfamily. Considering the second question, Freudenthal et al. (1992), Kristkoiz (1992) and Kalthoff (2006) have shown that the Melissiodontinae and Paracricetodontinae are different clades that do not have more in common than that they are both included in the Muridae.

Early diversification of the Melissiodontinae was also suggested by the allocation of *Edirnella kempeni* de Bruijn et al. 2003, from the late Eocene of eastern Turkey, to the Melissiodontinae on the basis of similarity in shape and morphology of its second and third upper and lower cheek teeth with those of the uncontested melissiodontine *Melisiodon bernlochensis* Hrubesch, 1957*.*

The presence of several melissiodontine species differing in the size of the cheek teeth, the height of the ridges relative to the cusps and in minor details of the microstructure of the enamel of the lower incisor in some of our Serbian localities came as a complete surprise (Table 1). The smaller species will be included in a new genus while the larger will tentatively be allocated to *Edirnella.* The uncertainty about the latter allocation is due to the unfortunate situation that, apart from one worn m2 (plate 7, Fig. 5 in Ünay-Bayraktar 1989), the lower dentition of the type species of *Edirnella* and the M1 of the two Serbian species is not known. The m2, m3, M2, and M3 that will be allocated to cf. *Edirnella a*re very similar to those of *Melissiodon.* However, the anterior part of the m1 (described and figured below) is very different.

*Mogilia* nov. gen.

**Type species:** *Mogilia miloshi* nov. sp.

**Type locality:** *Zvonce.*

**Age:** Eocene.

**Derivatio nominis:** After the old Slav word ‘Mogila’, meaning a site where something lies buried.

**Included species:** *Mogilia miloshi* nov. sp*.* and *Mogilia lautus* nov. sp.

**Diagnosis:** *Mogilia* species have low-crowned cheek teeth with slender, but relatively high, cusps and a moderately to very complex network of low irregular ridges. The anterocone of the M1 is broad and situated on the central longitudinal axis of the occlusal surface. The small retracted anteroconid of the m1 is lower than the protoconid and metaconid. The anterior outline of the m1 is rounded and the anterosinusid of the m2 and m3 is wide as in all Melissiodontinae. The m3 has approximately the same length as the m1. The molars lack a basal ring of lamellar enamel. The outer surface of the thin enamel of the lower incisor is either smooth or shows a set of indistinct tangential ridges. The PI consists of transverse HSB with prism parallel IPM that make an angle of ~ 30° with the normal on the EDJ. The PE consists of radial enamel.

**Differential diagnosis:** *Mogilia* differs from *Melissiodon* in having lower-crowned cheek teeth with lower, more irregular, ridges. The almost symmetrical anterocone complex consisting of three cusps is situated on the central longitudinal axis in *Mogilia*, but has a labial position in *Melissiodon.* The lingual part of the anteroconid of the m1 is connected to the protoconid in *Mogilia* as well as in *Melissiodon*, but the anterior outline of the m1 of these genera is quite different because a second, labially situated, anteroconid cusp developed in *Melissiodon.*

*Mogilia* differs from *Edirnella* by its more complex dental pattern and the structure and the central position of the anterocone complex of the M1. This complex consists of three cusps: a parastyl, the true anterocone and a cusp situated close to the lingual border between the anterocone and the protocone (= the *Melissiodon* cusp of Ünay-Bayraktar 1989). In *Edirnella*, the anterocone has a labial position; it is situated on a straight line with the metacone and paracone. The *Melissiodon* cusp is not really part of the anterocone complex and the parastyl is absent. In *Mogilia* the anteroconid of the m1 is a small cusp that is connected to the protoconid, while it is developed as a cingulum in *Edirnella kempeni.* The m1 of the type species of *Edirnella* (*E. sinani*) is unfortunately not known.

**Fig. 3:**
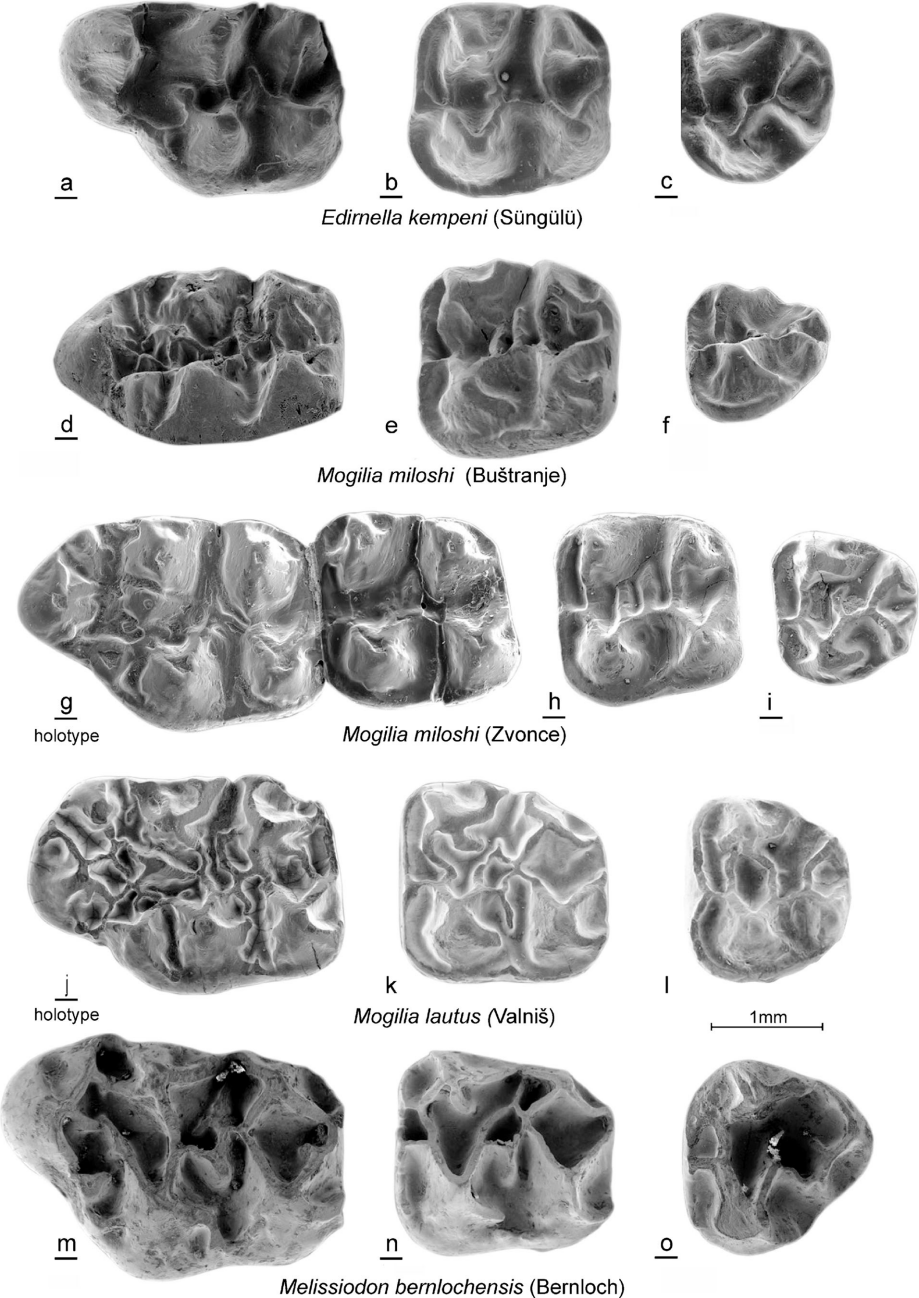
*Edirnella kempeni* casts from Süngülü (type locality). **a** M1. **b** M2. **c** M3*. Mogilia miloshi* nov. sp. from Buštranje (code 031). in **d** M1 (BUS-501). **e** M2 (BUS-511). **f** M3 (BUS-531)*. Mogilia miloshi* nov. sp. from Zvonce, (type locality, code 036). **g** M1–M2 (ZV-505, holotype). **h** M2 (ZV-518). **i** M3 (ZV-525). *Mogilia lautus* nov. sp. from Valniš (type locality, code 027). **j** M1 (VA-878, holotype). **k** M2 (VA-886). **l** M3 (VA-903)*. Melissiodon bernlochensis* from Bernloch (type locality). Collection Bayerische Staatssammlung, München. **m** M1 (nr.84). **n** M2 (nr.101). **o** M3 (nr.1530)

**Fig. 4:**
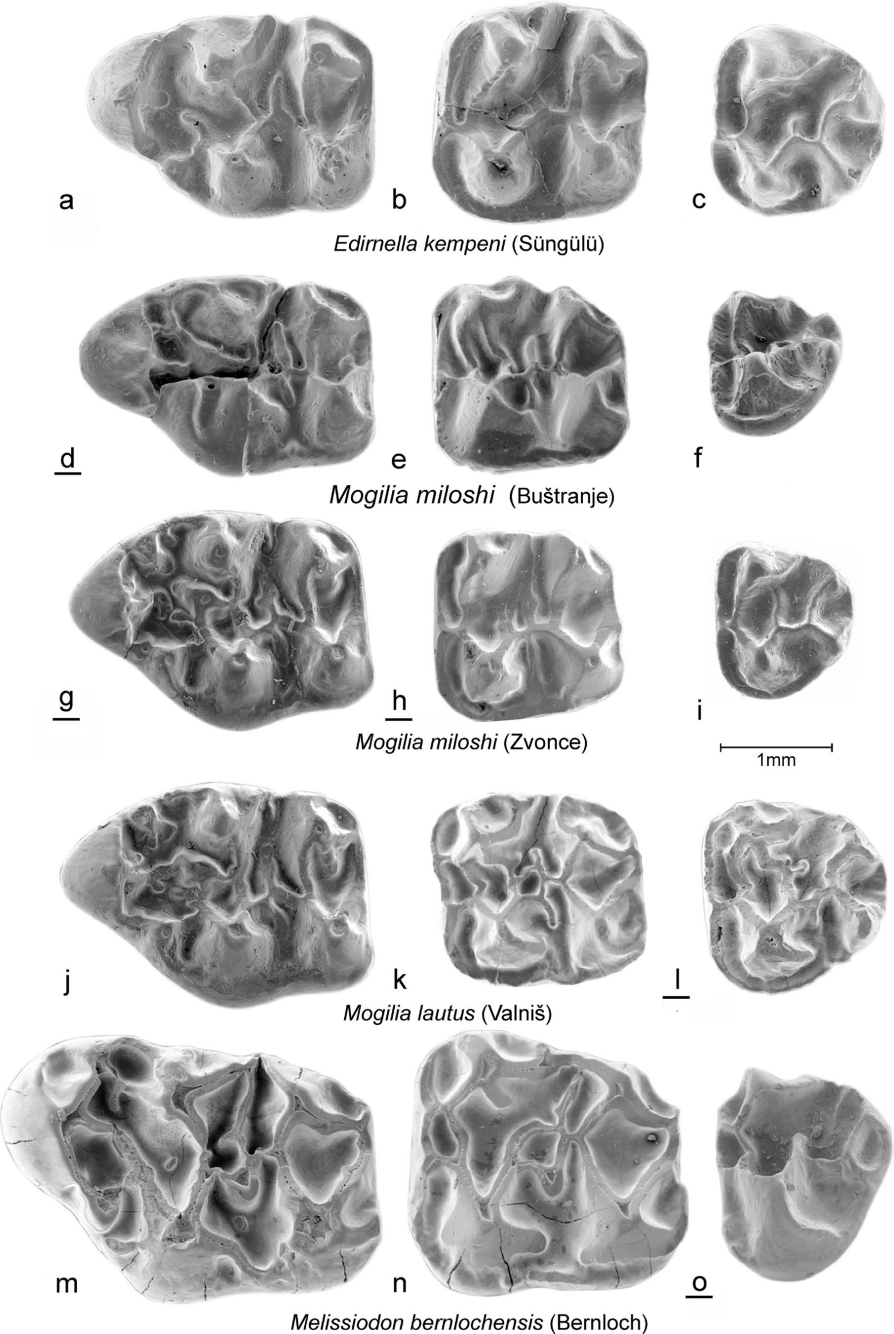
*Edirnella kempeni* from Süngülü (type locality). **a** M1. **b** M2. **c** M3*. Mogilia miloshi* nov. sp. from Buštranje, (code 031). **d** M1 (BUS-506). **e** M2 (BUS-512). **f** M3 (BUS-532). *Mogilia miloshi* nov. sp. from Zvonce, (type locality, code 036). **g** M1 (ZV-507). **h** M2 (ZV-517). **i** M3 (ZV-521)*. Mogilia lautus* nov. sp. from Valniš (type locality, code 027). **j** M1 (VA-871). **k** M2 (VA-881). **l** M3(VA-913)*. Melissiodon bernlochensis* from Bernloch (type locality). Collection Bayerische Staatssammlung München. **m** M1 (nr.891). **n** M2 (nr.1529). **o** M3 (nr.107)

**Fig. 5:**
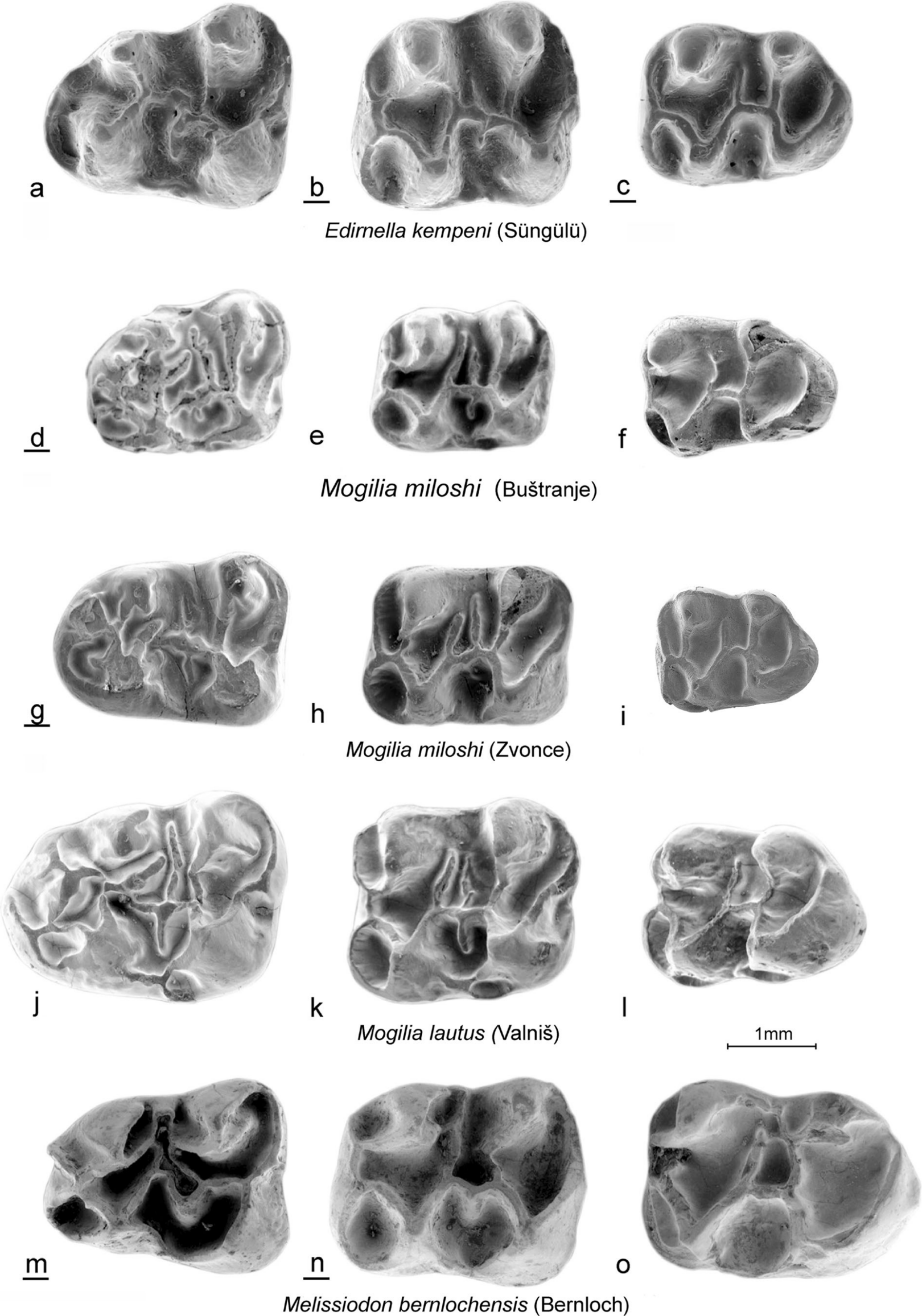
*Edirnella kempeni* from Süngülü (type locality). **a** m1. **b** m2. **c** m3. *Mogilia miloshi* nov. sp. from Buštranje (code 031). **d** m1 (BUS-556). **e** m2 (BUS-561). **f** m3 (BUS-581). *Mogilia miloshi* nov. sp. from Zvonce, (type locality, code 036). **g** m1 (ZV-546). **h** m2 (ZV-552). **i** m3 (ZV-561). *Mogilia lautus* nov. sp. from Valniš (code 027). **j** m1 (VA-9264). **k** m2 (VA-937). **l** (VA-956) Melissiodon bernlochensis from Bernloch (type locality). Collection Bayerische Staatssammlung, München). **m** m1 (nr.1536). **n** m2 (nr.65). **o** m3 (nr.25)

**Fig. 6:**
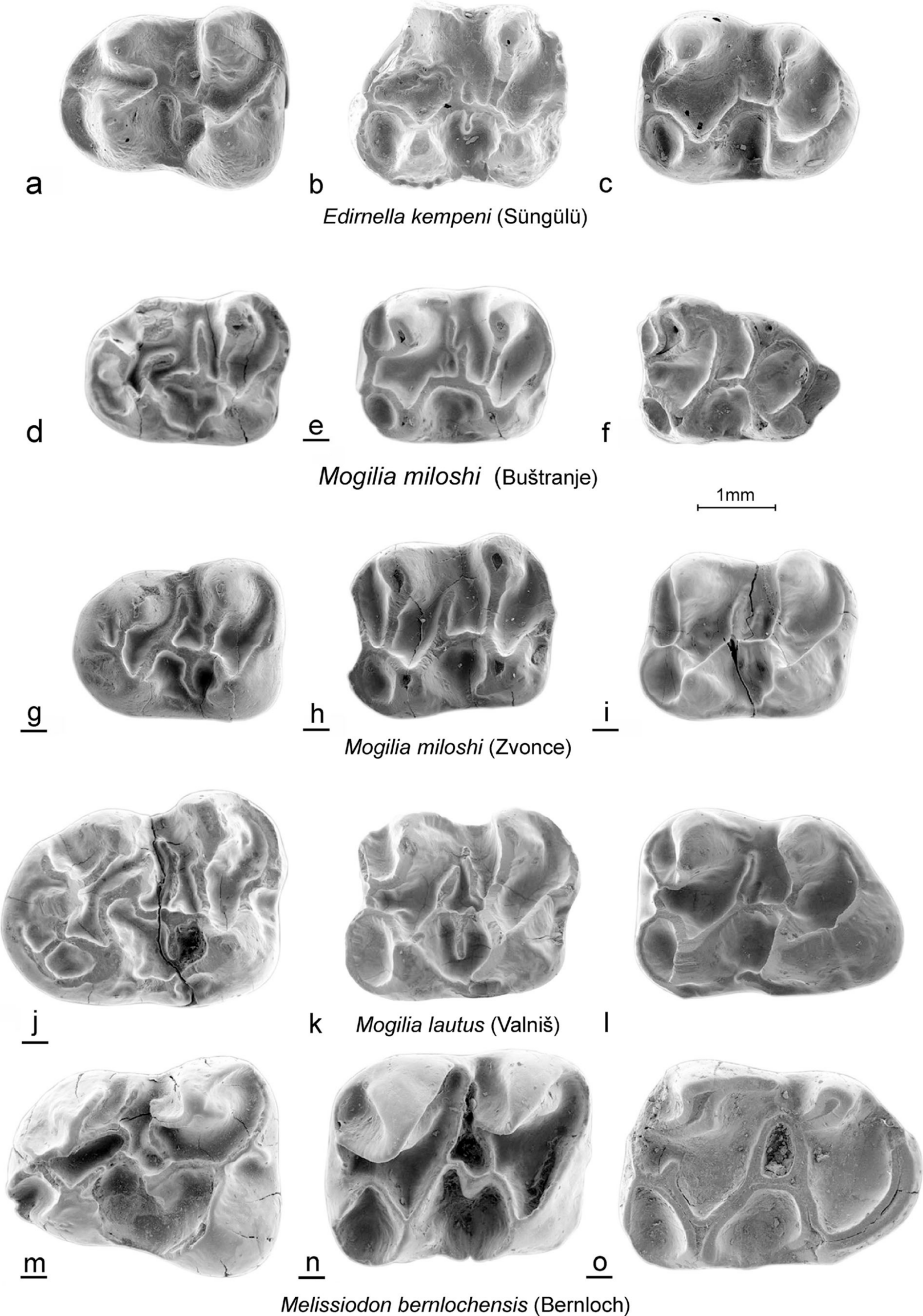
*Edirnella kempeni* from Süngülü (type locality). **a** m1. **b** m2. **c** m3. *Mogilia miloshi* nov. sp. from Buštranje, (code 031). **d** m1 (BUS-551). **e** m2 (BUS-571). **f** m3 (BUS-582). *Mogilia miloshi* nov. sp. from Zvonce, (type locality, code 036). **g** m1 (ZV-545). **h** m2 (ZV-557). **i** m2 (ZV-555). *Mogilia lautus* nov. sp. from Valniš (type locality, code 027). **j** m1 (VA-926). **k** m2 (VA-933). **l** m3 (VA-958). *Melissiodon bernlochensis* from Bernloch (type locality). Collection Bayerische Staatssammlung, München). **m** m1 (1536). **n** m2 (nr.65). **o** m3 (nr. 25)

*Mogilia miloshi* nov. sp.

(Figs. 3d–i, 4d–i, 5d–i, and 6d–i).

**Derivatio nominis:** This species is named after our friend and colleague Miloš Milivojević, one of the most successful fossil hunters of the eastern hemisphere.

**Type locality:** Zvonce (coordinates 42°55′54″- 22°34′43″).

**Age:** Eocene.

**Holotype:** Fragment of a right maxilla with M1-M2 (ZV-505) Fig. 3 g.

**Fig. 7:**
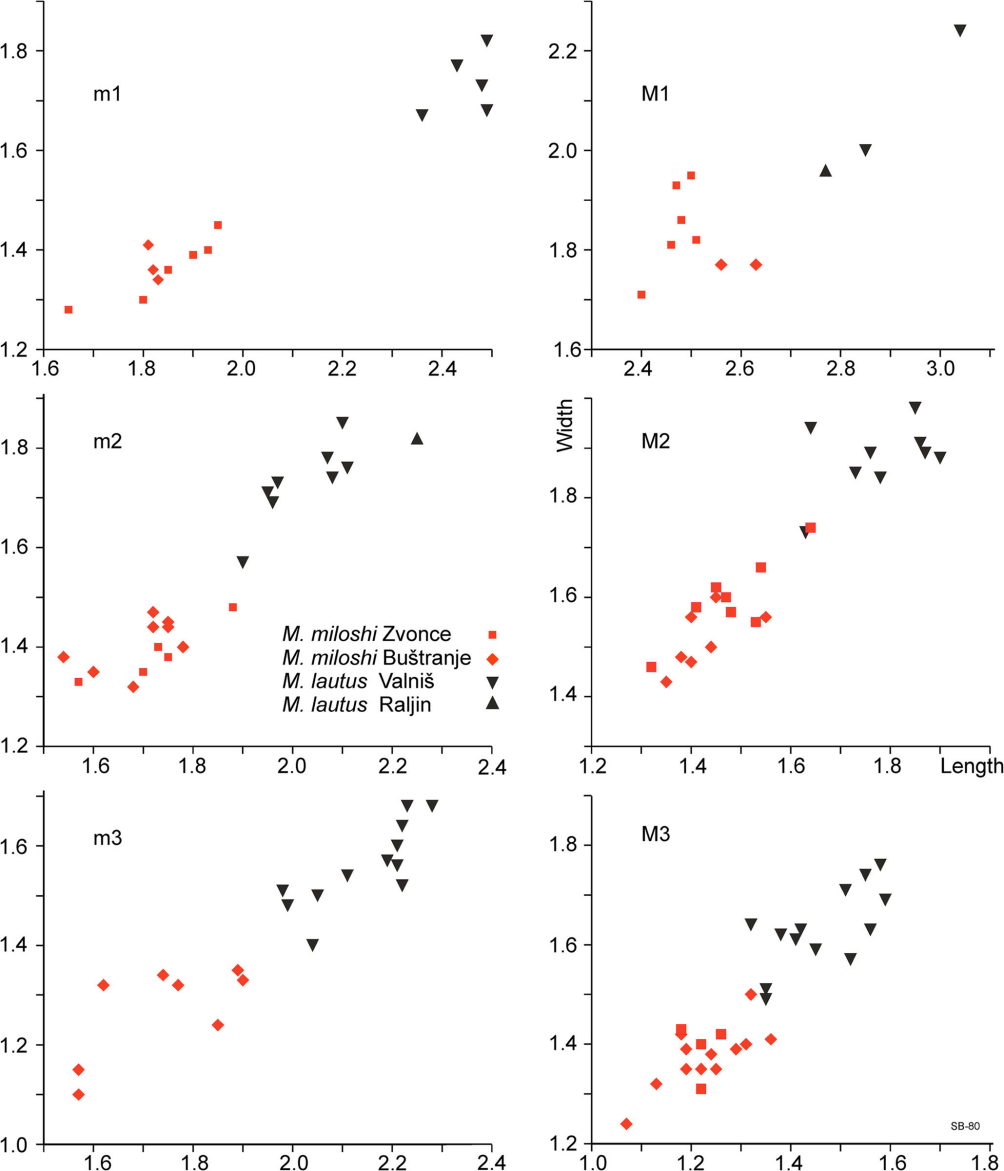
Length-width scatter diagrams of the cheek teeth of *Mogilia miloshi* from Zvonce (type locality) and Buštranje and *Mogilia lautus* from Valniš (type locality) and Raljin

**Material and measurements:** Tables 2 and 3, Fig. 7.

**Table 2:** Measurements of the cheek teeth of *Mogilia miloshi* from Zvonce

	Length (mm)			Width (mm)		
**Zvonce**	**Range**	**Mean**	**N**	**Mean**	**Range**	**N**
M1	2.40–2.50	2.47	6	1.85	1.70–2.00	6
M2	1.42–1.74	1.57	10	1.59	1.46–1.74	9
M3	1.18–1.26	1.22	4	1.39	1.31–1.43	4
m1	1.65–1.95	1.85	6	1.36	1.28–1.45	6
m2	1.57–1.88	1.73	5	1.40	1.33–1.48	6
m3	–	1.77	1	1.37	–	1

**Table 3:** Measurements of the cheek teeth of *Mogilia miloshi* from Buštranje

	Length (mm)			Width (mm)		
**Buštranje**	**Range**	**Mean**	**N**	**Range**	**Mean**	**N**
M1	2.56–2.63	2.60	2	1.77		2
M2	1.45–1.65	1.52	7	1.51	1.43–1.60	8
M3	1.07–1.36	1.24	19	1.36	1.24–1.50	21
m1	1.81–1.83	1.82	3	1.37	1.34–1.41	3
m2	1.40–1.78	1.68	11	1.41	1.32–1.47	10
m3	1.57–1.90	1.74	8	1.26	1.10–1.35	8

Upper dentition from the type locality shown in Figs. 3g–i and 4g–i.

Lower dentition from the type locality in Figs. 5g–i and 6g–i.

Other localities with *Mogilia miloshi*: Buštranje (late Eocene).

**Diagnosis:** Small species of *Mogilia*. Labial cusps of the upper cheek rounded, not strongly laterally compressed and ridges subordinate to the cusps. The anteroconid of the m1 is very small and, in the majority of the specimens, connected to the protoconid by a short oblique anterolophid.

**Differential diagnosis:** *Mogilia miloshi* differs from *M. lautus* by its smaller size and simpler dental pattern of the M1 and m1. Moreover, the labial cusps of the upper cheek teeth are not laterally compressed as in *M. lautus.*

### Description of the type material

All upper cheek teeth have three roots. In the M1, the anterocone proper is situated on the central longitudinal axis of the occlusal surface. Its size and height are about the same as in the four main cusps. The anterocone complex consists of the anterocone and a parastyl on the labial border and the *Melissiodon* cusp on the lingual border of the occlusal surface. A peculiar feature shared by all M1 is the small, but well delimited, cusp that sits on the longitudinal axis behind the anterocone. It is not clear whether this cusp is the homologue of the protoconule or a neoformation. Among the many low, irregular, ridges connecting the cusps, remnants of the, more or less transverse, protoloph, metalophule, mesoloph and posteroloph and of the longitudinal ridge can be detected.

The occlusal surface of the M2 is about square as in all melissiodontines. The labial arm of the anteroloph is long and reaches the antero-labial base of the paracone. The lingual arm of the anteroloph is even longer and continues as a cingulum that reaches the base of the hypocone. The protolophule 1 and the metalophule are transverse and connected to the anterior arms of the protocone and hypocone. A complete protolophule 2 is present in 5 out of 10 specimens; in the others, this ridge is incomplete and developed as a second mesoloph. The length of the mesoloph itself shows strong individual variation. In only 1 out of 10 specimens it reaches the labial border of the occlusal surface. The posteroloph descends sharply from the hypocone to the metacone.

The anterior part of the M3 is very similar to that in the M2. The posterior part of the M3 is reduced and shows the V- pattern that characterises most melissiodontine M3. The lingual arm of the anteroloph continues as a cingulum around the protocone and reaches the reduced hypocone. The protolophule 1 is connected to the anterior arm of the protocone. The length of the mesoloph shows much variation, but this ridge never reaches the labial border of the occlusal surface.

The lower cheek teeth have two roots. The m1 has a tiny anteroconid; it is not a true cusp in the majority of the specimens but the end of the anterior arm of the protoconid. Antero-labially of this ‘anteroconid’ there is an anterosinusid in four out of five specimens. The metalophulid 1 is directed obliquely forward and connects to the anterior arm of the protoconid. The strong posterior arm of the protoconid almost reaches the lingual border of the occlusal surface. An irregular, sometimes incomplete mesolophid is present in three out of five specimens. The hypolophulid is transverse or directed slightly forward and connects to the anterior arm of the hypoconid. The long posterolophid is connected to the postero-lingual slope of the entoconid.

The m2 has a long straight lingual arm of the anterolophid that is separated from the metaconid by a notch, while the shorter labial branch encloses the anterosinusid. The parallel, slightly forwards directed, metalophulid and hypolophulid connect to the anterior arms of the protoconid and hypoconid. The posterior arm of the protoconid is long. The mesolophid is long in two out of six, short in two out of six and absent in two out of six specimens. The long posterolophid is connected to the postero-lingual base of the entoconid.

The morphology of the single m3 from Zvonce available (Fig. 5i) is very similar to that of the m2. The metalopulid and hypolophulid are transverse and connect to the anterior arms of the protoconid and hypoconid. The posterior arm of the protoconid is long, but the mesolophid is absent.

Enamel structure of the lower incisor and the cheek teeth

The outer enamel surface of the lower incisor is either smooth (Buštranje) or shows an indistinct set of tangential ridges (Zvonce). The PI of the thin enamel (~ 30 mu) consists of transverse HSB with prism parallel IPM that make an angle of about 30° with the normal on the EDJ (Fig. 9). The PI of the specimen from Zvonce is about four times the thickness of the radial enamel of the PE, while the PI of the specimen from Buštranje is only slightly thicker than the PE. The enamel of the cheek teeth consists of radial enamel all the way to the base of the crowns (P-type, von Koenigswald 2004).

Comparison of the Mogilia miloshi cheek teeth from Buštranje and Zvonce

The teeth from Buštranje assigned to *M. miloshi* are metrically as well as morphologically very similar to those from Zvonce. The ridges of the few M1 available from Buštranje are somewhat higher and these teeth have a ridge connecting the ‘protoconule’ to the parastyl, a cusp that is less developed than in the M1 from Zvonce. The ridges of the M2 from Buštranje seem also to be slightly higher than in the specimens from Zvonce and the paracone and metacone are more laterally compressed. The morphology of the M3 from both localities overlaps. The ridges of the lower cheek teeth from Buštranje are slightly higher than in the ones from Zvonce and the anteroconid of the m1 is connected to the metaconid by the metalophulid in three out of four specimens. The m2 from the two localities differ sharply in the configuration of the metalophulid. In the six m2 from Zvonce the metalophulid connects labially with the anterior arm of the protoconid, while in the 11 specimens from Buštranje, the metalophulid and the anterior arm of the protoconid are connected to the anterolophid separately. The latter configuration is seen in most *Melissiodon* species as well as in *Edirnella kempeni*. The differences between the two samples of *M. miloshi* teeth observed above are in our opinion insufficient to define separate species. This is especially so because not all tooth positions would then allow identification to the species level. However, the morphological differences strongly suggest that Buštranje specimens are more derived and thus may be the younger of the two localities. Whether or not this age difference is at the basis of the sharp difference in composition between the rodent associations from these localities too (de Bruijn et al. in press) remains to be demonstrated.

*Mogilia lautus* nov. sp.

(Figs. 3j–l, 4j–l, 5j–l and 6j–l).

**Derivatio nominis:** *Lautus* has two meanings in Latin. The first is ‘well washed’ and the second is ‘‘luxurious’, both qualifications apply to the *Mogilia* teeth that will be described below.

**Holotype:** M1 dex. from Valniš (VA-878), Fig. 3j (shown reversed).

**Type locality: **Valniš.

**Age:** Early Oligocene.

**Other localities with**: *Mogilia lautus* Strelac-1, Raljin (both not illustrated).

**Age:** Early Oligocene.

**Material and measurements:** Tables 4 and 5, Fig. 7.

**Table 4:** Measurements of *Mogilia lautus* from Valniš

	Length (mm)			Width (mm)		
**Valniš**	**Range**	**Mean**	**N**	**Mean**	**Range**	**N**
M1	2.67–3.04	2.85	3	2.12	20.0–22.4	2
M2	1.73–2.00	1.88	9	1.88	1.73–1.98	9
M3	1.32–1.59	1.46	13	1.63	14.9–1.76	13
m1	2.36–2.49	2.45	5	1.78	1.67–2.02	6
m2	1.95–2.11	2.02	9	1.74	1.57–1.85	10
m3	1.98–2.28	2.14	12	1.56	1.40–1.68	12

**Table 5:** Measurements of *Mogilia lautus* from Strelac-1 and Raljin

	Length (mm)			Width (mm)		
**Strelac-1**	**Range**	**Mean**	**N**	**Mean**	**Range**	**N**
M3		1.59	1	1.66		1
**Raljin**						
m2		2.25	1	1.82		1

**Diagnosis:** *Mogilia lautus* is of medium size with upper cheek teeth that show an intricate pattern of low ridges. Among these is an exceptionally well-developed entomesoloph. The broad anterocone complex of the M1 is situated on the longitudinal axis of the occlusal surface. The unicuspid anteroconid of the m1 has a retracted position and a low cingulum at its anterior slope. A prominent extra cusp is present directly postero-labially of the metaconid.

**Differential diagnosis**: *Mogilia lautus* is larger than *M. miloshi* and the dental patterns of the upper teeth and the lower m1 are much more intricate than in that species. The elaborate structure of the anteroconid complex of the m1 of *M. lautus* is different from the small, simple anteroconid in the m1 of *M. miloshi.*

### Description of the type material

In the M1, the anterocone proper is situated on the central longitudinal axis of the occlusal surface. The equally large ‘parastyl’ and *Melissiodon* cusp are connected with the anterocone and with each other by a complex array of rather low ridges in which the smaller protoconule participates The pyramidal *Melissiodon* cusp, protocone and hypocone are situated on a straight line and so are the parastyl, paracone and metacone.

The M2 has a long lingual branch of the anteroloph; it continues as a lingual cingulum that reaches the hypocone. The labial branch is long also, but ends at the paracone in the majority of the specimens. The four main cusps, which have anterior as well as posterior arms, are much higher than the ridges connecting them. The metacone is more laterally compressed than the paracone. The long entomesoloph reaches the lingual border in three out of nine specimens.

The M3 has a long lingual branch of the anteroloph; it continues as a low cingulum along the lingual border of the occlusal surface. In some M3, this cingulum reaches the hypocone, in others, it is separated from that cusp by a notch. The labial branch is connected to the base of the paracone. An entomesoloph is present in 11 out of 13 specimens. The protolophule 1 connects the paracone to the protocone and the well-developed mesoloph forms a protolophule 2 in the majority of the M3.

In the m1, the configuration of the anterior part is most unusual because the anteroconid proper is not situated close to the anterior border, but has an internal position, while the end of the posterior arm of the protoconid has formed a cusp which is situated directly postero-labially of the metaconid. As far as we know, there is no other murid known with a cusp in this position. The mesolophid and ectomesolophid are long. The weak hypolophulid connects the entoconid with the anterior arm of the hypoconid.

In the m2, the metalophulid and the anterior arm of the protoconid reach the anterolophid separately. The protoconid and the labial branch of the anterolophid enclose an oval anterosinusid, a feature shared by most Melissiodontinae. The posterior arm of the protoconid, the mesolophid and the ectomesolophid are well developed in all 10 m2.

The anterior portion of the m3 is very similar to that of the m2. A long posterior arm of the protoconid is present in all 12 m3, but the mesoloph is absent in 6 out of 12 specimens. The hypolophulid inserts on the anterior arm of the hypoconid.

### Discussion

The m2 from Raljin is somewhat larger than the ones from the type locality of *M. lautus*, while it is morphologically intermediate between *M. lautus* and cf. *Edirnella* nov. sp. 2 from Strelac-1. In spite of an effort undertaken in 2015 to enlarge the collection from Raljin, we did not find any more complete melissiodontine teeth, so the assignation of this single m2 remains uncertain.

The teeth in the associations of *Mogilia* from Zvonce, Buštranje and Valniš show in this sequence a trend towards increase in size and complexity of the dental pattern. Since the samples from Buštranje and Zvonce are small, the difference in stage of evolution between these associations is for most tooth positions not clear. However, the metalophulid in the m2 from Zvonce connects to the anterior arm of the protoconid (the original murid configuration), while it bends forward and connects with the anterolophid in the specimens from Buštranje (the derived configuration). Although we have no independent age control that the locality of Zvonce is older than Buštranje, we interpret the observed difference between the *Mogilia* associations that way. Our working hypothesis is that *M. miloshi* and *M. lautus* have an ancestor-descendant relationship.

*Edirnella* Ünay-Bayraktar, 1989

#### **Included species:**

*Edirnella kempeni*, *Edirnella sinani,* cf. *Edirnella* nov. sp. 1 from Buštranje, cf. *Edirnella* nov. sp. 2 from Strelac 1.

The few specimens and fragments included in cf. *Edirnella* differ in size, cf. *Edirnella* nov. sp. 1 being larger than cf. *Edirnella* nov. sp. 2. The m1 of cf. *Edirnella* are different from those of *Mogilia* but more like the m1 of *E. kempeni*; hence, our assignation to cf. *Edirnella.* Unfortunately the m1 of the type species *E. sinani i*s not known, thus some uncertainty will remain in the allocation of species to the genus.

cf. *Edirnella* nov. sp. 1.

(Figs. 8d–g).

**Fig. 8:**
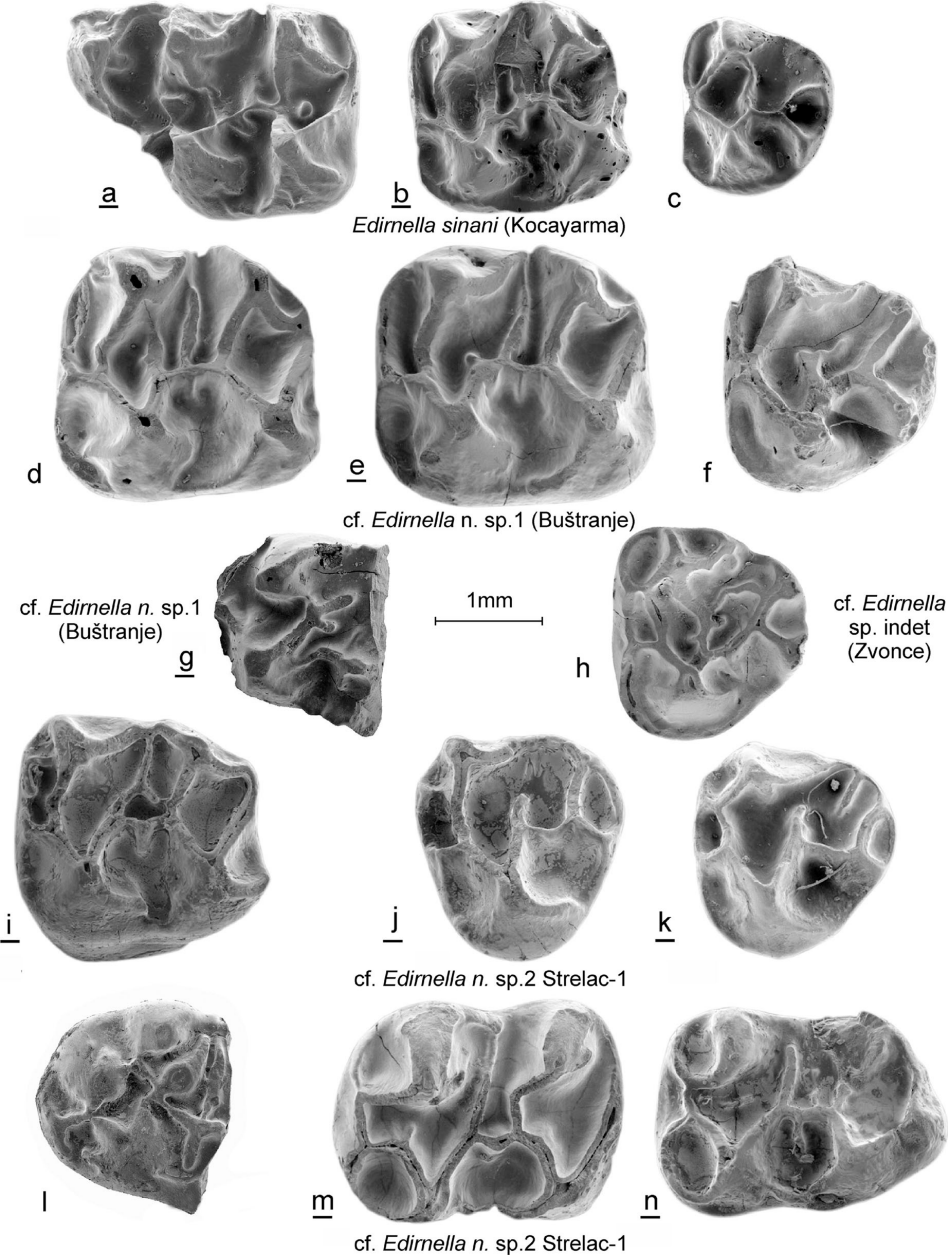
*Edirnella sinani* from Kocayarma (type locality). **a** M1. **b** M2. **c** M3. cf. *Edirnella* nov. sp. 1 from Buštranje (code 031). **d** M2 (BUS-591). **e** M2 (BUS-593). **f** M3 (BUS-596). **g** Anterior part of m1 (BUS-599). cf. *Edirnella* sp. from Zvonce (code 036). **h** (ZV-587). cf. *Edirnella* nov. sp. 2 from Strelac-1 (code 024). **i** M2 (STR1–302). **j** M3 (STR1–308). **k** M3 (STR1–310). **l** anterior part of m1 (STR1–311). **m** m2 (STR1–315). **n** m3 (STR1–318)

**Locality** Buštranje.

**Age:** Late Eocene.

**Dental characters:** The morphology of these few upper teeth is very similar to the type material of *E. sinani*, but they are much larger. The M2 has three roots.

**Remark:** The M2 and M3 of cf. *Edirnella* nov. sp. 1 resemble those of *Melissiodon* in the shape of the cusps and the height of the ridges, but the associated anterior part of an m1 shows more similarity with that tooth in *Mogilia* than in *Melissiodon* (see Figs. 5, 6 and 8).

**Material and measurements:** Only the M2, M3 and a damaged m1 are available.

Table 6 Measurements of cf. *Edirnella* nov. sp. 1 from Buštranje.

**Table 6:** Measurements of cf. *Edirnella* n. sp. 1 from Buštranje

	Length (mm)			Width (mm)		
**Buštranje**	**Range**	**Mean**	***N***	**Mean**	**Range**	***N***
M2	2.42–2.77	2.55	3	2.29	2.17–2.38	3
M3	–	1.92	1	2.13	–	1
m1	–	2.37	1	–	–	–

### Description

**The M2:** The lingual and labial branch of the anteroloph have approximately the same length. The transverse protolophule 1 connects to the anterior arm of the protocone. The protolophule 2 is not attached to the metacone. The mesoloph is long in two specimens and of medium length in two others. The protocone and hypocone are V-shaped while the high paracone and metacone bear burgee-shaped posterior spurs.

**The M3:** The anterior half of the M3 is very similar to the M2. The hypocone and metacone are reduced and incorporated into the ridges.

The m1. The damaged centrally placed anteroconid was probably small and low. The posterior arm of the protoconid is separated from the metaconid by a shallow notch and ends free similar to the configuration in *M. miloshi.*

The enamel of the lower incisor and the cheek teeth

**Fig. 9:**
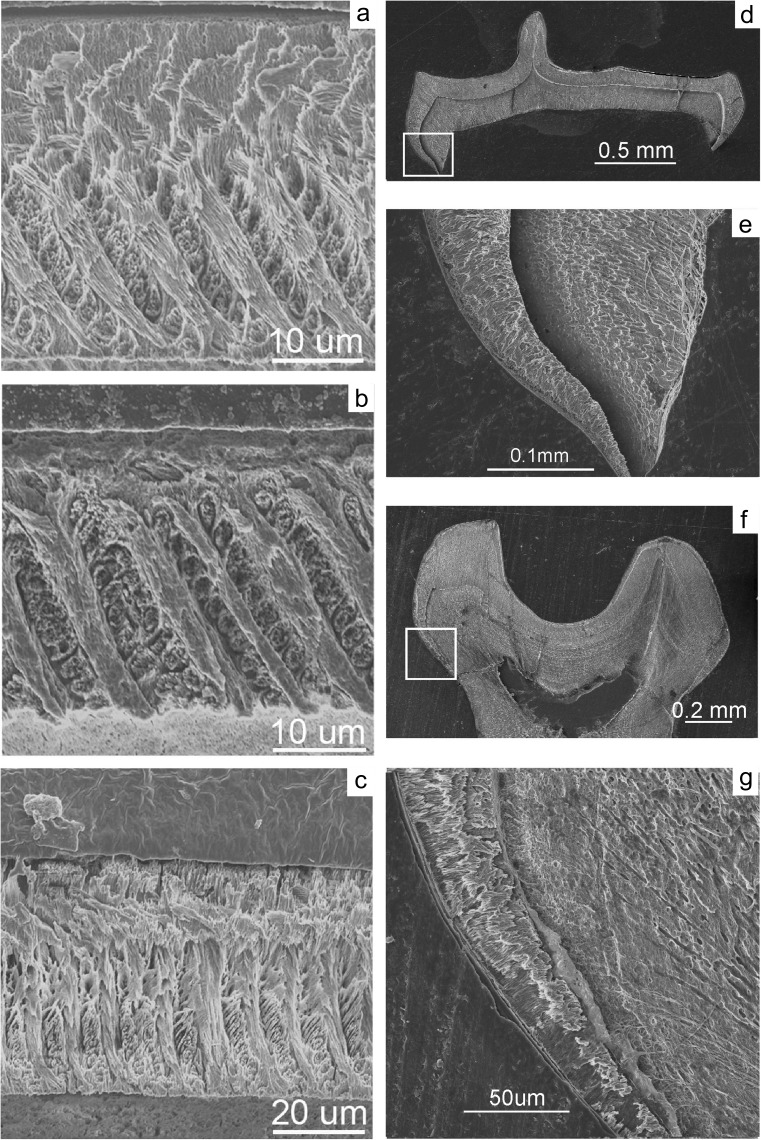
**a** Longitudinal section of the lower incisor enamel of *Mogilia miloshi* nov. sp. from Buštranje. **b** Longitudinal section of the lower incisor enamel of *Mogilia miloshi* nov. sp. from Zvonce. **c** Longitudinal section of the lower incisor enamel of *Edirnella* nov. sp. 1 from Buštranje. **d-e** Transverse section of the m2 of *Mogilia miloshi* nov. sp. from Buštranje. **f-g** Transverse section of the m2 of *Edirnella* nov. sp. 1 from Buštranje

The outside of the enamel of the lower incisor shows an indistinct set of tangential ribs. The PI of the thin (~ 50 mu) enamel consists of transverse HSB with prism-parallel IPM (Fig. 9). The angle between the HSB and the normal on the EDJ is ~ 0°. The thickness of the PE is about 30% of that of the PI. The PLEX is thin or absent (Fig. 9c).

The cross section of a fragment of an m2 (Fig. 9f, g) shows that there is no ring of lamellar enamel at the base of the cheek teeth of cf. *Edirnella* nov. sp. 1.

cf. *Edirnella* nov. sp. 2.

(Fig. 8i–n).

**Localities:** Strelac-1 and Strelac-2.

**Age:** Early Oligocene.

**Dental characters:** The morphology of the M2 and M3 is very much like those teeth of *Edirnella* and *Melissiodon*, but the configuration of the anterior part of the m1 resembles that of *Mogilia* (Fig. 6g, j). The teeth of cf. *Edirnella* nov. sp. 2 are intermediate in size between those of cf. *Edirnella* nov. sp. 1 and *Edirnella sinani.*

**Material and measurements:** The M1 and the lower incisor are not available.

Table 7. Measurements of cf. *Edirnella* nov. sp. 2 from Strelac-1 and -2. The Strelac-2 m1 is rolled.

**Table 7:** Measurements of cf. *Edirnella* n. sp. 2 from Strelac-1 and -2

	Length (mm)			Width (mm)		
**Strelac-1**	**Range**	**Mean**	***N***	**Mean**	**Range**	***N***
M2	–	2.15	1	1.97	1.89–2.05	2
M3	1.58–1.79	1.66	5	1.82	1.66–1.93	5
m2	2.42–2.62	2.52	2	1.89	1.84–1.94	2
m3	–	2.66	1	1.86	–	1
Strelac-2						
M3 sup	–	1.63	1	1.75	–	1

### Description

The morphology of the three-rooted M2 of *Edirnella* nov. sp. 2 resembles that of the M2 of *Melissiodon bernlochensis* in detail (Figs. 3n, 4n and 8i). The anteroloph is low and has a double connection to the transverse protolophule 1 and is weaker than in *Edirnella* nov. sp. 1. Characteristic is the small pit that is enclosed by the entoloph, the posterior arm of the protocone and the mesoloph. This pit seems to be precluded by the configuration of low ridges in the central part of the M2 of *Mogilia lautus* from Valniš (Fig. 3k).

The five M3 from Strelac-1 show considerable individual variation in the differences in degree of reduction of the posterior part of the occlusal surface. A peculiar characteristic that all these teeth share is the double connection between the protoloph and the anteroloph. The taxonomic value of this character is uncertain; it occurs also in the M3 of cf. *Edirnella* sp. indet. From Zvonce (Fig. 8h) and in the M3 of *M. bernlochensis* (Figs. 3o and 4o), but lacks in the M3 of *Edirnella* nov. sp. 1 from Buštranje (Fig. 8f).

The single m1 available from Strelac-1 consists of an anterior part only (Fig. 8l). This anteroconid complex resembles the configuration seen in the m1 of *Mogilia lautus* (Figs. 5j and 6j) in having a retracted anteroconid lingually which is connected by a short anterolophulid to a smaller, more ridge-shaped and a labially situated anteroconid cusp. Another characteristic shared by these two species is the development of an extra cusp near, or at the end of the posterior arm of the protoconid. The morphology of the rolled specimen from Strelac-2 is as described above.

The m2 shows a protoconid and hypoconid that are V-shaped and thus modified as in *Melissiodon.* The anterosinusid is wide. The metalophulid and the anterior arm of the protoconid reach the anterolophid separately. The posterior arm of the protoconid continues as a long thin mesolophid all the way to the lingual border of the occlusal surface as in the m2 of *Melissiodon bernlochensis* (Figs. 5n and 6n). The hypolophulid connects to the anterior arm of the hypoconid.

The long m3 has a dental pattern, slightly reduced, that is almost identical to that of the m2 (Fig. 8m, n).

cf. *Edirnella* sp. indet.

(Fig. 8h).

**Locality:** Zvonce.

**Age:** Late? Eocene.

**Material and measurements** 1 M3 (length 1.75 mm, width 1.93 mm).

### Description

The single M3 from Zvonce is much too large to allocate it to *Mogilia miloshi* from that locality. Its morphology, showing a double connection between the protoloph and the anteroloph, and size are similar to the M3 of *Edirnella* nov. sp. 2 from the late Oligocene site Strelac-1. Since the M3 of Melissiodontinae are very much alike this specimen cannot be identified to the species level.

## The evolutionary history of the Melissiodontinae

Until Ünay-Bayraktar (1989) assigned her much disputed genus *Edirnella* from the early Oligocene of Turkish Thrace to the Melissiodontinae the geographic range of this, then monogeneric, subfamily remained restricted to Europe where it is considered to be an immigrant from the East. The first occurrence of *Melissiodon* is in Bernloch (MP23), a site in southern Germany East of the Rhine Graben, while the first appearance west of the Rhine Graben is from localities assigned to MP24 (Russel et al. 1982). The stratigraphic range of the genus *Melissiodon* in Europe is from late early Oligocene (MP23) to well into the early Miocene (MN4). It is the only murid which range straddles the Cricetid Vacuum in south western Europe. Nine species have been formally named (Hrubesch 1957), which seems, considering the stability of their highly derived dental morphology, too many. Other than *Edirnella sinani* from the Thrace basin (MP25, Ünay-Bayraktar 1989) there are only two records of Melissiodontinae from outside of Europe: *E. kempeni* from the late Eocene of Süngülü (Lesser Caucasus; de Bruijn et al. 2003) and *Melissiodon* sp. from the Oligo/Miocene transitional interval of Kargı-2 in central Anatolia (de Bruijn et al. 2013).

The discovery of the diverse array of Melissiodontinae from the late Eocene and early Oligocene of southeastern Serbia described above thus increases our knowledge of the early history of the subfamily substantially. We interpret the rapid consequent specialisation of the morphology of the chewing apparatus of the Melissiodontinae as an adaptation to feeding on small invertebrates.

It shows that its, presumably Asian, ancestor colonised the Serbian-Macedonian land area during the early or middle Eocene. That is, during the dawn of the origin of the Muridae, thus after the split up of the Muridae and Dipodidae, and before the ‘Grande Coupure’, so the Melissiodontinae seem to be a very early branch of the Muridae. This conclusion is supported by the occurrence of two species in the late Eocene locality Buštranje that not only differ in the size and morphology of their cheek teeth, but also in details of the microstructure of their incisor enamel (Fig. 9). This radiation into several species probably occurred on or near the Serbian-Macedonian land area.

## References

[CR1] Bruijn, H. de, Ünay, E., Saraç, G. & Yilmaz, A. (2003). A rodent assemblage from the Eo/Oligocene boundary interval near Süngülü, Lesser Caucasus, Turkey. In López-Martínez, N., Peláez-Campomanes, P. & Hernández Fernández, M. (eds.). *En torno a Fósiles de Mamíferos: Datación, Evolución y Paleoambiente. Coloquios de Paleontología, Volumen Extraordinario no 1. En honor al dr. Remmert Daams*. 47–76.

[CR2] Bruijn, H. de, Ünay, E. and Hordijk, K. (2013). A review of the Neogene succession of the Muridae and Dipodidae from Anatolia, with special reference to taxa known from Asia and/or Europe. In: Wang, A., Flyn, L.J. & M. Fortelius (eds). *Fossil Mammals of Asia: biostratigraphy and chronology*. Columbia University Press, p. 556–582.

[CR3] Bruijn, H. de, Marković, Z., Wessels, W., Milivojević, M., & van de Weerd, A. A. (in press). Rodent faunas from the Paleogene of south-east Serbia. *Palaeobiodiversity and Palaeoenvironments*. 10.1007/s12549-017-0305-0. PMC6417383 30956713

[CR4] Freudenthal, M., Lacomba J. I., & Sacristán, M. A. (1992). Classification of European Oligocene cricetids. *Revista Espagnola Paleontología, Extra Volume*, 49–57.

[CR5] Freudenthal M, Hugueney M, Moissenet E (1994). The genus *Pseudocricetodon* (Cricetidae, Mammalia) in the upper Oligocene of the province of Teruel (Spain). Scripta Geologica.

[CR6] Hrubesch, K. (1957). Zahnstudien an tertiären Rodentia als Beitrag zu deren Stammesgeschichte: Über die Evolution der Melissiodontidae, eine Revision der Gattung *Melissiodon*. Abhandlungen Bayerische Akademie Wissenschaften, *Mathematisch*-*Naturwissenschaftliche Klasse* Neue Folge, 83, 1–100.

[CR7] Kalthoff DC (2000). Die Schmelzmikrostructur in den Incisiven der hamsterartigen Nagetiere und anderer Myomorpha (Rodentia, Mammalia). Palaeontographica A.

[CR8] Kalthoff DC (2006). Incisor enamel microstructure and its implications to higher level systematics of Euroasian Oligocene and early Miocene hamsters (Rodentia). Palaeontographica A.

[CR9] Koenigswald, W. von (2004). The three elementary types of schmelzmuster in rodent molars and their occurrence in the various rodent clades. *Palaeontographica A*, 270, 95–132.

[CR10] Kristkoiz A (1992). Zahnmorphologische und schädelanatomische Untersuchungen an Nagetieren aus dem Oberoligozän von Gaimersheim (Süddeutschland). Abhandlungen Bayerische Akademie Wissenschaften, Mathematisch-Naturwissenschaftliche Klasse. Neue Folge.

[CR11] Russel, D. E., Hartenberger, J. L., Pomerol, C., Şen, S., N. Schmidt-Kittler & M. Vianey, Liaud, M. (1982). Mammals and stratigraphy: the Paleogene of Europe. *Palaeovertebrata, Memoire extraordinaire*, 1–77.

[CR12] Schaub S (1920). *Melissiodon* n. gen., ein bisher übersehener oligocäner Muride. Senckenbergiana.

[CR13] Schaub S (1925). Die Hamsterartigen Nagetiere des Tertiärs und ihre lebenden Verwandten. Abhandlungen Schweizerischen Pälaeontologische Gesellschaft.

[CR14] Ünay-Bayraktar E (1989). Rodents from the middle Oligocene of Turkish Thrace. Utrecht Micropaleontological Bulletins, Special Publications.

